# Clinical and immunological features in patients with neuroimmune complications of COVID-19 during Omicron wave in China: a case series

**DOI:** 10.3389/fimmu.2024.1499082

**Published:** 2024-12-18

**Authors:** Siyin Gong, Bo Deng, Hai Yu, Xiang Zhang, Wenbo Yang, Xiangjun Chen

**Affiliations:** ^1^ Department of Neurology, Huashan Hospital, Fudan University and Institute of Neurology, Fudan University, National Center for Neurological Disorders, Shanghai, China; ^2^ Department of Neurological Diseases, The Second Affiliated Hospital, Chongqing Medical University, Chongqing, China

**Keywords:** COVID-19, Guillain-Barre syndrome, myelitis, autoimmune encephalitis, encephalitis

## Abstract

**Purpose:**

This study aimed to present clinical and immunological features in patients with neuroimmune complications of COVID-19 during Omicron wave in China.

**Methods:**

Patients with neuroimmune complications associated with COVID-19 were retrospectively analyzed in Huashan Hospital from December 2022 to April 2023, during the widespread prevalence of Omicron variants in China. Demographic information, symptoms, electrophysiological findings, cerebrospinal fluid(CSF) test results and immunological markers, Magnetic Resonance Imaging(MRI) characteristics, treatment strategies and outcomes of these patients were reviewed and analyzed.

**Results:**

A total of 53 cases of neuroimmune complications were included, with 7 cases of non-immune complications taken as controls. Neuroimmune complications comprised: 7 cases of Guillain-Barre syndrome/chronic inflammatory demyelinating polyneuropathy, 11 cases of spinal meningitis/myelitis, 2 cases of neuromyelitis optica spectrum disorder, 2 cases of myelin oligodendrocyte glycoprotein antibody-associated disease, 1 case of acute disseminated encephalomyelitis, 10 cases of autoimmune encephalitis, 17 cases of other encephalopathy/encephalitis and 3 cases of cerebellitis. SARS-CoV-2 was only detected in the CSF sample of one neuroimmune complications patient. CSF-restricted oligoclonal bands were detected in 11.1% (5/45) of neuroimmune patients, but absent in non-immune cases (0.0%, 0/5). Autoantibody testing identified specific antibodies in 26.5%(13/49) of neuroimmune cases and 0.0% (0/5) of non-immune cases. Glucocorticoids or intravenous immunoglobulins were administered as first-line treatments for all neuroimmune cases (100%, 53/53), whereas only 42.8% (3/7) of non-immune cases received these therapies. A baseline modified Rankin scale (mRS) score of 3 or above was present in the majority of both neuroimmune cases (96.2%, 51/53) and non-immune cases (71.4%, 5/7). At the end of a follow-up period, independent functional outcomes at day-90 with an mRS score below two were observed in a significant proportion of both neuroimmune cases (77.4%, 41/53) and non-immune case(71.4%, 5/7).

**Conclusion:**

The manifestations of neuroimmune complications of COVID-19 are diverse and can manifest with severe neurological deficits early in the course of the disease. The detection of immunological markers (such as autoantibody and oligoclonal bands) and immunotherapies can help to improve the prognosis of COVID-19 related neuroimmune complications.

## Introduction

As of 17 March 2024, severe acute respiratory syndrome coronavirus type 2 (SARS-CoV-2) has resulted in over 774 million confirmed infections and 7 million deaths due to coronavirus disease-2019 (COVID-19) worldwide (1). During the initial outbreak of COVID-19 in Wuhan, neurological complications were frequently identified in hospitalized patients, which involved the central nerve system (CNS), peripheral nerve system (PNS) and skeletal muscles (2). Studies have indicated that the incidence of neurological complications in hospitalized COVID-19 populations in China, Europe, and the United States exceeds one-third (2–4). Even in hospitalized COVID-19 patients devoid of major manifestations in the CNS, SARS-CoV-2 can lead to subtle neuro-axonal damage, as indicated by the elevation of blood neurofilament light chain protein (5). On the other hand, in the post-infectious stage, postural orthostatic tachycardia syndrome (POTS) and other long COVID-19 symptoms are receiving increasing recognition in patients with COVID-19 (6).

In the early phases of the COVID-19 pandemic, the Chinese government adopted strict measures to curb the spread of the virus. Nevertheless, with the advent of the highly contagious Omicron variant and the alteration of the epidemic control policy on December 7, 2022, Omicron variants began to spread extensively throughout China (7, 8). Neurological symptoms were reported in 22.3% of pediatric COVID-19 inpatients, a prevalence significantly higher than the 8% observed in the pre-Omicron period (9). SARS-CoV-2 impairs the nervous system through several pathophysiological mechanisms, including an overly active immune response causing inflammation and damage (10, 11), disruption of the blood-brain barrier (12), and indirect effects attributed by systemic illness and hypoxia (13). Additionally, the direct viral invasion of the brain can result in neurological symptoms and complications (14). Nevertheless, the presence of SARS-CoV-2 spike protein or viral particles in neurons and other brain cells is limited (15), and the low levels of detectable virus do not correlate with the histopathological alterations (16). Since the outbreak of the COVID-19 pandemic, neuroimmune diseases, such as autoimmune encephalitis(AIE), other encephalopathy/encephalitis, myelitis and Guillain-Barre syndrome(GBS) have been increasingly reported. In these cases, the neurological system is impaired by generalized neuroinflammation with the trafficking of immune cells, cytokines, and antibodies into the brain and the activation of microglia, which is exacerbated by antibody production, including antibodies against to SARS-CoV-2 and autoantibodies (15).

Currently, studies reporting neuroimmune complications associated with COVID-19 lack comprehensive detection of immunological markers, such as neural autoantibodies, oligoclonal bands, lymphocyte subsets and cytokines, as well as evaluation of complete assessment of the effect of immunotherapy. The aims of the present article are to present the clinical and immunological features in patients with neuroimmune complications of COVID-19 during Omicron wave in China.

## Methods

### Participants

Patients with neurological complications associated with COVID-19 were retrospectively analyzed in Huashan Hospital from December 2022 to April 2023. We defined COVID-19 associated neurological complications as meeting the following criteria:(1) an acute onset of neurological symptoms within one month after the diagnosis of COVID-19,(2) proof of COVID-19, confirmed by reverse transcriptase-polymerase chain reaction (RT-PCR) and antigen test using oropharyngeal or nasopharyngeal swab sample. Patients were excluded if they had any coexisting condition that could explain the neurological symptoms. Among them, non-immune complication cases were used as controls.

### Data collection

Patients’ clinical information was retrieved from electronic medical records, face-to-face or telephone interviews. Demographic information, symptoms, laboratory data, immunological markers, electrophysiological findings, and magnetic resonance imaging(MRI) characteristics were collected.

Laboratory data encompassed cerebrospinal fluid (CSF) white blood cell and protein analysis, as well as CSF microbiological testing, including next-generation sequencing(NGS). Electrophysiological tests comprised electroencephalogram(EEG), nerve conduction velocity(NCV), and somatosensory evoked potentials(SEP). MRI sequences were as follows: T1-weighted imaging (T1WI), T2-weighted imaging (T2WI), Fluid-attenuated inversion recovery (FLAIR), and T1-weighted imaging with contrast enhancement (T1WI with contrast), In some cases, Diffusion-weighted imaging (DWI) and Susceptibility-weighted imaging (SWI) sequences were also included. Immunological markers included: CSF or serum autoantibodies (with the antibody panel selected based on specific neurological manifestations), oligoclonal bands, lymphocyte subsets, and cytokines.

Treatment strategies and outcomes of these patients were reviewed. We used the modified Rankin scale (mRS) to evaluate patients’ disease severity and outcomes during 90-day follow-ups.

### Statistical analysis

Statistical analysis was performed with SPSS, version 20.0 (SPSS, Chicago, IL). Categorical variables were presented as percentages, and differences between two groups were compared with the χ^2^ test. Quantitative variables with normal distribution were presented as mean ± standard deviation (SD), and Student’s-t test was employed for comparison between two groups. Quantitative variables with non-normal distribution were presented by median (25th and 75th centiles), and Mann-Whitney U test was utilized between two groups. Statistical significance was regarded as present if the 2-tailed p value was less than 0.05.

## Results

A total of 53 patients were diagnosed with neuroimmune complications associated with COVID-19 in this case series, 7 case of non-immune disorders were identified as controls. Neuroimmune complications comprised: 7 cases of GBS/chronic inflammatory demyelinating polyneuropathy (CIDP), 11 cases of spinal meningitis/myelitis, two cases of neuromyelitis optica spectrum disorder (NMOSD), 2 cases of myelin oligodendrocyte glycoprotein antibody-associated disease (MOGAD), 1 case of acute disseminated encephalomyelitis (ADEM), 10 cases of AIE (according to previous diagnostic criteria (17, 18), including specific antibody-mediated AIE, autoimmune limbic encephalitis, and antibody-negative AIE), 17 cases of other encephalopathy/encephalitis (according to Consensus Statement of the International Encephalitis Consortium (19)) and 3 cases of cerebellitis. Meanwhile, the non-immune complications included 3 cases of ischemic stroke, 2 cases of pontine and extrapontine myelinolysis, 1 case of cerebral venous thrombosis (CVT), and 1 case of anxiety disorder.

**Figure 1:**
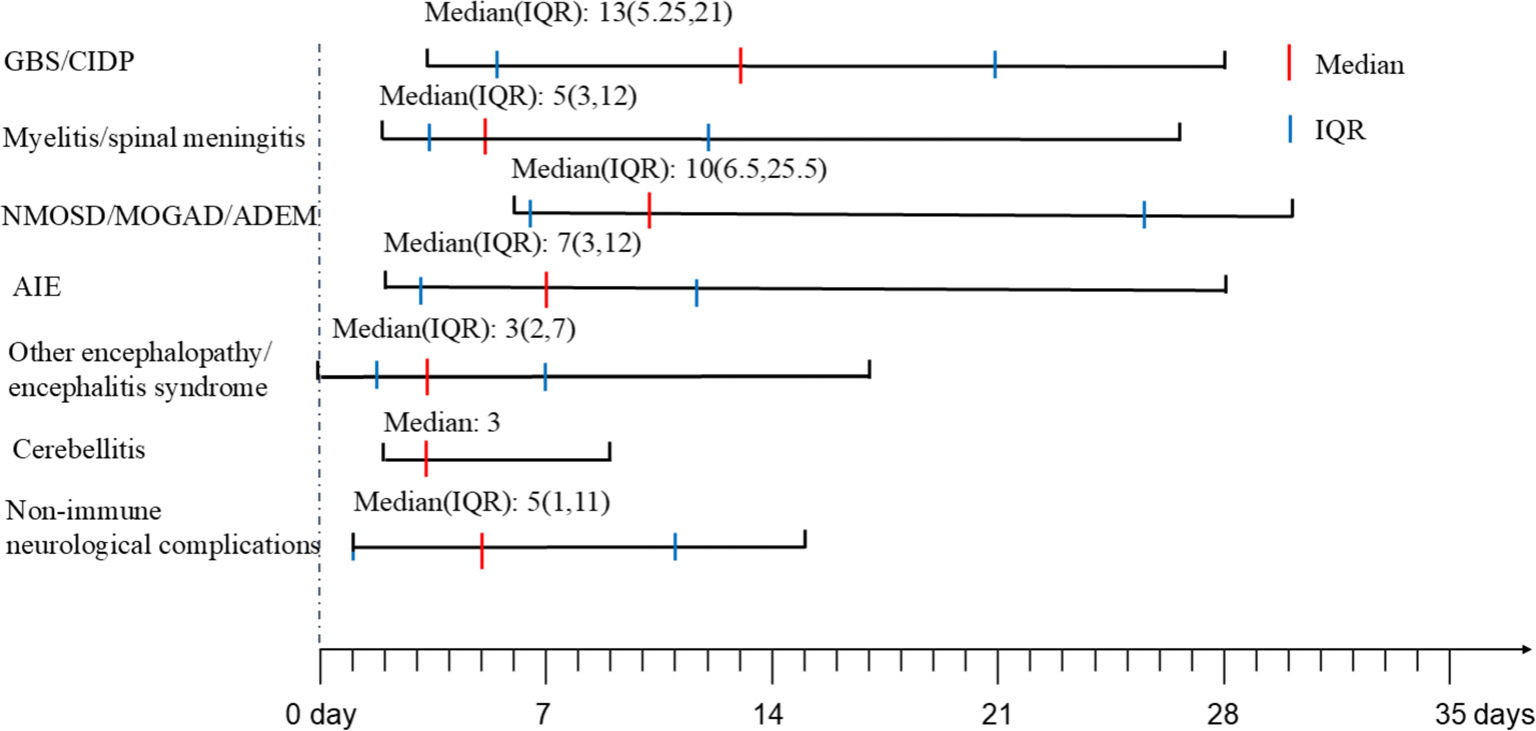
The onset time of neurological complications after COVID-19. GBS, Guillain-Barre Syndrome; CIDP, chronic inflammatory demyelinating polyneuropathy; NMOSD, neuromyelitis optica spectrum disorders; MOGAD, myelin oligodendrocyte glycoprotein antibody-associated disease; ADEM, acute disseminated encephalomyelitis; AIE, autoimmune encephalitis; IQR, interquartile range.

All cases in this series presented symptoms of COVID-19 with varying severities. Fever was the most uniformly reported symptom, followed by respiratory manifestations, like cough and sore throat. Pneumonia and pulmonary embolism were identified in 15.1% (8/53) vs. 14.2% (1/7) and 1.9% (1/53) vs. 14.2% (1/7) of neuroimmune and non-immune cases, respectively. Gastrointestinal symptoms, such as diarrhea or vomiting were found in 5.7% (3/53) vs. 28.6% (2/7) of cases. RT-PCR or antigen tests of SARS-CoV-2 using naso- or oropharyngeal swab samples were positive in all patients. Among them, 90.1%(20/22) vs. 100.0% (2/2) of patients in neuroimmune and non-immune cases were serologically positive for COVID-19. The interval between COVID-19 infection and onset of neurological complications was showed in **Figure 1**. The median days between COVID-19 infection and onset of neurological complications of neuroimmune and non-immune cases were showed in **Table 1**.

**Table 1:** Immunological markers and prognosis of neuroimmune and non-immune neurological patients.

	Neuroimmune patients (n=53)	Non-immune neurological patients (n=7)
Age (year)	43.30 ± 21.6	43.7 ± 16.8
Sex (male)	39.6% (21/53)	42.9% (3/7)
Onset time (day)	6 (3, 12)	5 (1, 11)
SARS-COV2 IgG	90.1% (20/22)	100.0% (2/2)
B cell increased	63.8% (30/47)	20.0% (1/5)
OCB positive	11.1% (5/45)	0.0% (0/5)
Auto-antibodies	26.5% (13/49)	0.0% (0/5)
Cytokines increased	48.6% (17/35)	50.0% (1/2)
Baseline mRS (≥3)	96.2% (51/53)	71.4% (5/7)
90d mRS (≤2)	77.4% (41/53)	71.4% (5/7)

OCB, oligoclonal bands; mRS, modified Rankin scale.

### GBS/CIDP

In 42.9% (3/7) of inflammatory peripheral neuropathy cases, chronic or recurrent presentations(CIDP) were observed, while 57.1% (4/7) exhibited acute onset (GBS). GBS or CIDP manifestations emerged subsequent to COVID-19, with a median onset delay of 13 days (interquartile range (IQR): 5.25 to 21 days). The patients ranged in age from 28 to 62 years, with 57.1% (4/7) being male. The majority of patients (85.7%, 6/7) manifested paraparesis or tetraparesis, and 71.4% (5/7) reported sensory symptoms. Cranial nerve involvements were noted in 42.9% (3/7) of cases. Respiratory failure occurred in one patient (14.3%). Based on clinical and electrophysiological findings, 57.2% (4/7) were diagnosed with GBS, and the remainders with CIDP. Acute inflammatory demyelinating polyneuropathy (AIDP) and acute motor-sensory axonal neuropathy (AMSAN) were both present in 50% of GBS cases.

CSF analysis conducted on all seven patients demonstrated classical albuminocytological dissociation (cell count ≤ 5/µl with elevated CSF proteins) in 42.9% (3/7). Oligoclonal bands were examined in five patients, and none of them were found positive. Serum interleukin-6 (IL-6) was elevated in 33% (1/3) of cases tested. All patients underwent autoantibody testing in serum or cerebrospinal fluid, with 42.9% of the patients testing positive. Anti-GD1b (serum and CSF) and anti-GM1 immunoglobulin G (IgG) (serum) were positive in one patient with AMSAN, anti-NF186 IgG (serum and CSF) was positive in one patient with AIDP, and anti-GM4 IgG, anti-MAG IgM (serum) and anti-GD3, anti-GD2 IgG (CSF) were positive in one patient with CIDP.

All patients presented with severe functional impairments before intervention, evidenced by a baseline mRS score of 3 or above. Intravenous immunoglobulin (IVIg) was the first-line therapy for all seven patients. Notably, one patient with respiratory failure received both plasma exchange(PLEX) and high-dose methylprednisolone(HDMP). Additionally, a subset of three patients diagnosed with CIDP was treated with a combination of glucocorticoids and immunosuppressants, specifically tacrolimus or cyclophosphamide. Outcome measures at 90 days post-treatment indicated significant functional recovery (mRS ≤ 2) in 85.8% (6/7) of the patients.

### Myelitis/spinal meningitis/NMOSD/MOGAD/ADEM

In cases involving the spinal cord, neurological manifestations emerged with a median of 8.5 days (IQR: 4.25 to 12.75 days) after the onset of COVID-19 symptoms. The patients’ ages ranged from 13 to 77 years, with 37.5% (6/16) being male. The predominant clinical presentations included paraplegia/quadriplegia and sensory alterations, often accompanied by autonomic dysfunction affecting the bowel and bladder control. Additionally, one patient with NMOSD experienced hiccups, while another with MOGAD suffered from vision loss and neck pain. And a patient with ADEM exhibited tremors and an unsteady gait.

CSF analysis revealed pleocytosis in 54.5% (6/11) of myelitis/spinal meningitis cases, while 45.5% (5/11) presented with normal CSF findings. Pleocytosis was identified in 50% (1/2) of NMOSD cases, while all CSF samples from MOGAD and ADEM patients showed pleocytosis, with one MOGAD case demonstrating a notably elevated white blood cell (WBC) count (>100 cells/mm³). Elevated CSF total protein was noted in 50.0% (8/16) of cases. Specific oligoclonal bands in CSF were detected in 23.1% (3/13) of cases (one for ADEM and two for myelitis). Autoantibodies tests in CSF or serum were performed in 93.8% (15/16) of cases, with 13.3% (2/15) of patients identified with anti-aquaporin-4 (AQP4) IgG, and 13.3% (2/15) of patients identified with anti-myelin oligodendrocyte glycoprotein(MOG) IgG. Cytokines in CSF or serum were tested in 81.3% (13/16) of cases, and increased CSF or serum cytokines were found in 38.5% (5/13) of cases.

**Figure 2:**
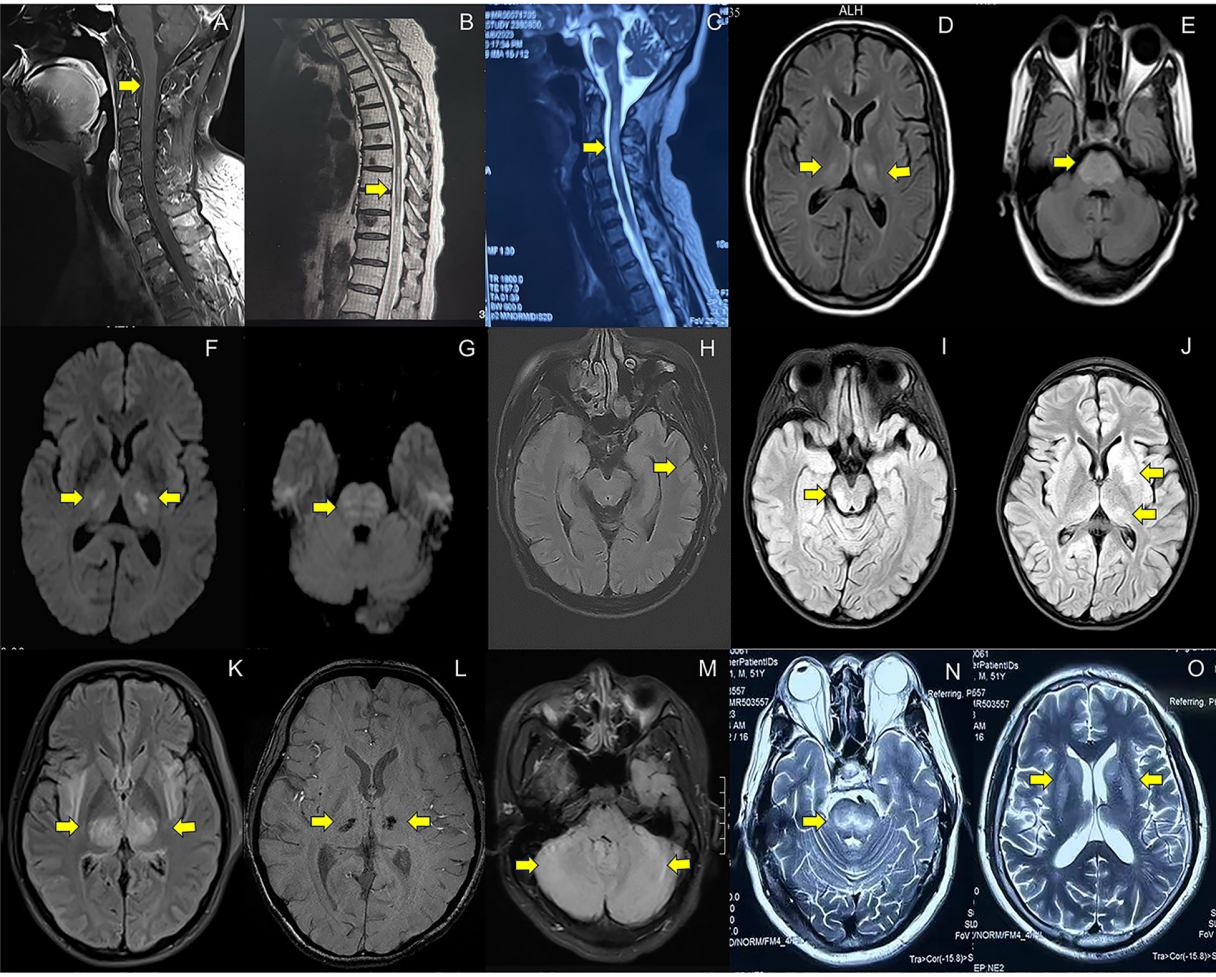
MRI imaging in patients with neurological complications associated with COVID-19. **(A)**. Abnormal enhancement of the cervical spinal cord meninges on T1-weighted imaging with contrast enhanced sequences (case 9, spinal meningitis); **(B)**. Longitudinally extensive hyperintensity lesions at T_8_-L_2_ level on T2WI(case 17, myelitis); **(C)**. Longitudinally extensive hyperintensity lesions at C_1-6_ level on T2-weighted imaging(T2WI) (case 1, neuromyelitis optica spectrum disorders); **(D–G)**. Symmetrical hyperintensity lesions in pons, bilateral hippocampus, thalamus, basal ganglia on Fluid-Attenuated Inversion Recovery (FLAIR) **(D, E)** and Diffusion-Weighted Imaging (DWI) **(F, G)** (case 39, other encephalopathy/encephalitis); **(H)**. Gyrus-like hyperintensity lesions with swelling cortex in left temporal lobe on FLAIR (case 32, limbic encephalitis); **(I, J)**. Hyperintensity lesions in pons, thalamus and basal ganglia on FLAIR (case 27, the overlapping syndrome of MOG-antibody disease and NMDAR encephalitis); **(K, L)**. Hyperintensity lesions in bilateral thalamus on FLAIR **(K)** and susceptibility-weighted imaging(SWI) detect hemorrhages in bilateral thalamus **(L)** (case 48, acute necrotizing encephalopathy); **(M)**. Hyperintensity lesions in bilateral cerebellum on FLAIR (case 52, cerebellitis); **(N, O)** Symmetrical hyperintensity lesions in bilateral basal ganglia and brainstem on T2WI (pontine and extrapontine myelinolysis). Detailed case information and numbers are available in the **Supplementary Materials**.

Spinal MRI was performed on all patients (16/16). Within the subgroup diagnosed with myelitis/spinal meningitis (11 cases), 27.3% (3/11) exhibited no detectable lesions in spinal cord on MRI. 18.2% (2/11) of cases demonstrated abnormal meningeal enhancement in the cervical spinal cord (**Figure 2A**) or conus medullaris. The majority (6/11) displayed T2WI hyperintensity lesions (**Figure 2B**), some with additional enhancement. For cases with NMOSD, MRI results consistently revealed longitudinally extensive transverse myelitis (LETM) (**Figure 2C**). Similarly, both patients with MOGAD presented spinal cord lesions, with one individual displaying T2WI/FLAIR hyperintensity in the left frontal lobe, cerebral peduncles, and bilateral limbic lobes.

The significant 93.8% (15/16) of patient presented with a baseline mRS score of 3 or higher prior to intervention. HDMP were the first-line treatments for 81.3% (13/16) of patients, with additional PLEX, IVIg or immunoabsorption (IA) therapy administered to 53.8% (7/13) of cases. All patients diagnosed with NMOSD and MOGAD were managed with various immunosuppressants, including mycophenolate mofetil (MMF), cyclophosphamide, or inebilizumab. Among those with myelitis/spinal meningitis, 54.5% (6/11) were treated with immunosuppressive agents. 56.3% (9/16) of the patients achieved an independent functional status (mRS ≤ 2) within 90 days.

### Autoimmune encephalitis and other encephalopathy/encephalitis

A total of 10 patients were diagnosed with AIE, including 3 cases of autoimmune limbic encephalitis, 2 cases of anti-N-methyl-D-aspartic acid receptor (NMDAR) encephalitis, 1 case of anti-glycine receptor(GlyR)1 encephalitis, 1 case of anti-gamma-aminobutyric acid type B receptor (GABA_b_R) encephalitis, 1 case of anti-Ri encephalitis, 1 case of the overlapping syndrome of MOG-antibody disease and NMDAR encephalitis (MNOS), and 1 case of antibody-negative AIE. Conversely, 17 patients were diagnosed with other encephalopathy/encephalitis, including 1 case of acute necrotizing encephalopathy (ANE).

**Figure 3:**
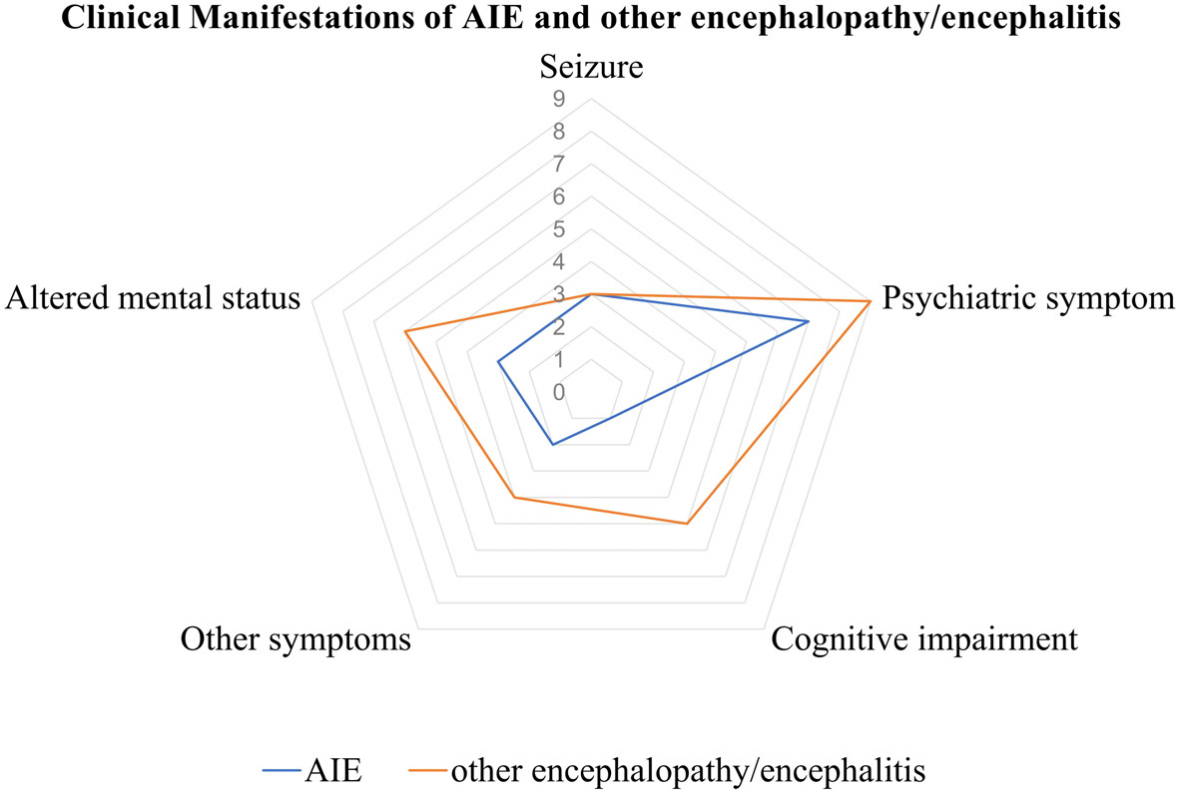
Clinical manifestations of AIE/other encephalopathy/encephalitis associated with COVID-19. AIE, autoimmune encephalitis.

Neurological manifestations emerged at a median of 7 days (IQR: 3 to 12 days) in AIE and 3 days (IQR: 2 to 7 days) in other encephalopathy/encephalitis patients subsequent to COVID-19 symptoms. The predominant clinical symptoms across AIE and other encephalopathy/encephalitis comprised psychiatric disorders, cognitive impairments, altered mental status and seizure (**Figure 3**). There were no significant differences in the clinical manifestations between the two groups of patients (**Table 2**). Notably, the patient with MNOS also exhibited decreased vision and an unsteady gait, and the patient with ANE was primarily characterized by fever and consciousness disorder.

**Table 2:** Clinical features of AIE and other encephalopathy/encephalitis patients.

	AIE (n=10)	Other encephalopathy/encephalitis (n=17)
Age (year)	37.6 ± 28.1	40.1 ± 23.6
Sex (male)	30.0% (3/10)	41.2% (7/17)
Covid-19 confirmed		
RT-PCR	80.0% (8/10)	52.9% (9/17)
Antigen test	20.0% (2/10)	52.3% (9/17)
Complicating disease		
Pneumonia	30.0% (3/10)	17.7% (3/17)
Pulmonary embolism	0.0% (0/10)	5.6% (1/17)
Hyperthyroidism	0.0% (0/10)	11.8% (2/17)
SARS-COV2 IgG	100.0% (6/6)	100% (4/4)
Onset time (day)	7 (3,12)	3 (2,7)
Clinical manifestation		
Fever	60.0% (6/10)	35.3% (6/17)
Psychiatric symptom	70.0% (7/10)	53.0% (9/17)
Altered mental status	30.0% (3/10)	35.3% (6/17)
Seizure	30.0% (3/10)	17.7% (3/17)
Cognitive impairment	10.0% (1/10)	29.4% (5/17)
Other	20.0% (2/10)	23.5% (4/17)
MRI abnormal	50.0% (5/10)	58.8% (10/17)
PET-CT		
FDG-PET abnormal	100.0% (7/7)	80.0% (4/5)
TSPO- PET abnormal	40.0% (2/5)	50.0% (2/4)
EEG abnormal	100.0% (7/7)	94.8% (15/16)
B cell increased	42.9% (3/7)	80.0% (12/15)
CSF		
WBC (/uL)	9 (1.0,13.5)	7 (1.0,16.5)
Total protein (mg/L)	436.0 (360.0,870.0)	540.0 (457.5,880.0)
NGS abnormal	50.0% (3/6)	14.3% (1/7)
OCB positive	25.0% (2/8)	0.0% (0/16)
Auto-antibodies*	60.0% (6/10)	0.0% (0/17)
Cytokines increased	50.0% (3/6)	77.8% (7/9)
HDMP	70.0% (7/10)	47.1% (8/17)
IVIg	90.0% (9/10)	70.6% (12/17)
PLEX	10.0% (1/10)	17.6% (3/17)
Mechanical ventilation	10.0% (1/10)	11.8% (2/17)
RTX or MMF*	40.0% (4/10)	5.9% (1/17)
Anti SARS-COV2	20.0% (2/10)	5.9% (1/17)
Baseline mRS (≥3)	90.0% (9/10)	100% (17/17)
90d mRS (≤2)	100.0% (10/10)	64.7% (11/17)

*P<0.05.

AIE, autoimmune encephalitis; RT-PCR, reverse transcription-polymerase chain reaction; MRI, magnetic resonance imaging; PET-CT, positron emission tomography computed tomography; FDG-PET, 18F-Fluorodeoxyglucose positron emission tomography; TSPO-PET, translocator protein positron emission tomography; EEG, electroencephalograph; CSF, cerebrospinal fluid; WBC, white blood cell; NGS, next generation sequencing; OCB, oligoclonal bands; HDMP, high-dose methyl prednisolone; IVIg, intravenous immunoglobulin; PLEX, plasma exchange; RTX, rituximab; MMF, mycophenolate mofetil; mRS, modified Rankin scale.

From all CSF samples obtained, pleocytosis was found in 55.6% (5/9) of AIE and 29.4% (5/17) of other encephalopathy/encephalitis cases, respectively. NGS was performed in 60% (6/10) of AIE and 47.1% (8/17) of other encephalopathy/encephalitis cases. Only one case with other encephalopathy/encephalitis was found SARS-CoV-2 (2 copies), with 5/µl WBC, while three AIE cases were found Epstein-Barr virus or herpes simplex virus 1 positive (low copies). CSF-restricted oligoclonal bands were more frequently identified in AIE cases, while increased CSF or serum cytokines, elevated total B cell proportion in serum were more frequently observed in other encephalopathy/encephalitis cases, even though there were no significant differences (**Table 2**). Detection of specific autoantibodies was performed in all cases. In AIE cases, the detected autoantibodies in CSF or serum included anti NMDAR IgG (2/10), anti GABA_b_R IgG (1/10), anti-Ri IgG (1/10), anti GlyR1 IgG (1/10), and anti NMDAR IgG was accompanied by anti MOG IgG (1/10).

All the patients underwent brain MRI examinations. 50.0% (5/10) of cases with AIE and 41.2% (7/17) of cases with other encephalopathy/encephalitis showed no abnormality. The typical images of other encephalopathy/encephalitis showed T2WI/FLAIR hyperintensity lesions in bilateral hippocampus, temporal lobes, thalamus or basal ganglia, and pons (**Figures 2D-G**), which were found in 23.5% (4/17) of other encephalopathy/encephalitis cases, while 40.0% (4/10) of AIE patients showed limbic involvement (**Figure 2H**), and the patient with MNOS presented FLAIR hyperintensity lesions in right optic nerve, cerebellum, pons and thalamus (**Figures 2I, J**). The images of ANE revealed the typical symmetric FLAIR hyperintensity and SWI hypointensity lesions in thalamus (**Figures 2K, L**). Notably, in MRI-negative cases which also had PET examinations, Fluorodeoxyglucose positron emission tomography(FDG-PET) or translocator protein (TSPO) PET were 100.0% (6/6) positive. The most common images showed decreased FDG uptake in bilateral cerebral cortex, increased FDG uptake in bilateral basal ganglia, mild elevation of TSPO binding in the medial aspect of temporal lobes. Only one case (3.7%) presented normal EEG, while the others showed slowing background with, increased delta or theta activities, and 20% (4/20) of cases also showed sharp waves or spike-and-slow waves.

Before treatment, 90.0% (9/10) of AIE and 100.0% (17/17) of other encephalopathy/encephalitis patients present with a baseline mRS score of 3 or higher. Glucocorticoids were the first-line treatments for all patients. Additionally, 90.0% (9/10) of AIE and 76.5% (13/17) of other encephalopathy/encephalitis cases received PLEX or IVIg therapy. More AIE than other encephalopathy/encephalitis cases (40.0% vs. 5.9%, p=0.047) underwent sequential treatment with MMF or rituximab (RTX). And more AIE than other encephalopathy/encephalitis patients (100.0% vs. 64.7%, P=0.057) achieved an mRS score of 2 or less in 90 days. The patient with ANE received additional rehabilitation and hyperbaric oxygen therapy, and showed significant improvement, with the most recent mRS score being 1.

### Cerebellitis

Three patients were diagnosed with cerebellitis, and neurological manifestations emerged at a median of 7 days (IQR: 3 to 12 days) subsequent to COVID-19 symptoms. The patients’ ages ranged from 18 to 59 years, with 66.6% (2/3) being male. The primary clinical symptoms were gait and speech ataxia. CSF analysis disclosed mild pleocytosis, and elevated total protein was observed in two-thirds (66.7%) of the cases. None of the patients had CSF-restricted oligoclonal bands or detectable autoantibodies in CSF and serum samples. One-third (33.3%) of the patients exhibited elevated serum cytokine levels (IL-1β, IL-5). Brain MRI findings included FLAIR hyperintensity lesions in the bilateral cerebellum (**Figure 2M**) in two out of the three cases, while the remaining case displayed a normal MRI. All patients initially presented with an mRS score of 3 or higher. Glucocorticoids were the initial treatment for all patients. By the 90-day follow-up, 66.7% (2/3) of the patients had achieved an mRS score of 2 or less.

### Non-immune neurological complications

A total of 7 non-immune neurological complications cases were identified during this period. Specifically, there were 3 cases of ischemic stroke, 2 cases of pontine and extrapontine myelinolysis, 1 case of CVT, and 1 case of anxiety disorder. The time interval between COVID-19 symptoms and the onset of neurological manifestations ranged from 1 to 15 days. The age range for affected patients was between 16 and 60 years old, with males accounting for approximately 71.4% (5/7) of the identified cases. Cerebrovascular events (ischemic stroke and CVT) characterized focal neurological deficits, while pontine and extrapontine myelinolysis manifested as cognitive impairment and psychiatric abnormalities. Anxiety disorder presented somatization disorder. Pontine and extrapontine myelinolysis brain MRI images exhibited symmetrically increased signal intensity on T2WI and FLAIR sequences, primarily affecting the central part of the pons (also known as “Trident Sign”), as well as regions beyond the pons such as the basal ganglia and thalami (**Figures 2N, O**).

Immunological markers and prognosis of non-immune cases were compared with neuroimmune cases in **Table 1**. 71.4% (5/7) of cases underwent autoantibody testing, yielding negative results for all cases. 71.4% (5/7) of patients were subjected to oligoclonal bands testing, which did not yield any positive findings. Lymphocyte subset testing was performed on 71.4% (5/7) of the patients, revealing an elevation in total B cells in only one patient (20% of cases have been screened for lymphocyte subset). Among the participants, 71.4% (5/7) had a baseline mRS score exceeding 3, while immunotherapy was administered to 42.8% (3/7) of the cases. A total of 71.4% (5/7) of the patients achieved an mRS score below 2 at the end of the 90-day period.

## Discussion

This study aims to describe the clinical and immunological characteristics, and prognosis of neuroimmune complications associated with COVID-19 during omicron wave in China. In our case series, we found that: (1) the neuroimmune complications associated with COVID-19, including GBS/CIDP, myelitis, spinal meningitis, NMOSD, MOGAD, ADEM, AIE, other encephalopathy/encephalitis and cerebellitis had a more marked non-infectious inflammatory profile rather than infectious profile, (2) oligoclonal bands, autoantibody assessments and PET-CT were helpful in evaluating neuroimmune complications associated with COVID-19, and (3) timely identification of the disease entities and initiation of immunotherapies(HDMP, IVIG and PE) may play a crucial role in enhancing prognosis.

The SARS-CoV-2 accesses the CNS by crossing the olfactory bulb (20) or breaching the blood-brain barrier through ACE2 receptor-mediated vascular damage (11, 21, 22). However, evidence of virus in CSF was rarely confirmed by PCR tests (23), and biopsy evidence of COVID−19 brains and spinal cord revealed inflammatory or necrobiotic changes without detectable virus (24, 25), suggesting limited direct viral presence. Para-infectious complications may be associated with the immune response to viral infections. Initiated by the innate immune system, this process involves the recognition of pathogens and the subsequent production of proinflammatory cytokines and chemokines (26). CSF proinflammatory cytokines, including IL-6, had been found markedly increased in adults with COVID-19 and neurological symptoms (27). Excessive cytokine release and autoimmune reactions, where the immune system mistakenly targeted CNS components similar to viral proteins, might cause CNS damage (28). Post-infectious complications may persist for weeks or months after the acute phase of infection. The adaptive immune system reacts subsequently, through which T cells directly eliminate virus-infected cells, and B cells generate antibodies specific to the pathogen circulating in the serum or present at mucosal surfaces (26). The occurrence of COVID-19-associated complications may be attributed to molecular mimicking between viral proteins and neuronal self-antigens (29).

During the COVID-19 pandemic, the incidence of GBS varied, with some studies showing an increase to 1.41 per 100,000 person-years from a pre-pandemic rate of 0.89 (30), while others reported a decrease (31), linked to fewer respiratory and gastrointestinal infections during lockdowns (32, 33). SARS-CoV-2 infections were linked with odds ratios of 6.30 for developing GBS (34). Further, a significant genetic interaction involving specific genes and the NOD-like receptor signaling pathway was identified (35). Additionally, a genetic predisposition related to SARS-CoV-2 infection notably increases the risk of NMO-IgG+ (OR = 5.512) (36). These findings underscore a complex relationship between COVID-19 and neuroimmune diseases, integrating epidemiological and genetic data.

The clinical manifestations, treatment strategies and prognosis of GBS (37, 38)/CIDP (39), myelitis (40), AIE (41, 42) and other encephalopathy/encephalitis (22, 23) were similar to those not associated with COVID-19. PCR for SARS-CoV-2 in CSF were negative in almost all cases examined (23, 40, 43), and CSF analysis reflected a non-infectious inflammatory process in most instances (23, 40, 44). Elevated cytokine levels were detected in the CSF or serum of some patients (23, 40, 43). Additionally, autoantibodies, including anti-GQ1b IgG, anti GM2 IgM/IgG, and anti GD1b IgM/IgG for GBS (43), anti-MOG, AQP4 IgG for myelitis, anti-NMDAR, MOG, glutamic acid decarboxylase(GAD), contact resistance protein related protein-2(CASPR2), voltage-gated potassium channels(VGKC), and glioma inactivation protein 1(LGI1) IgG for AIE (45), were present in parts of patients. These findings, derived from laboratory results and therapeutic outcomes, suggest that neurological complications associated with COVID-19 represent para-infectious or post-infectious diseases rather than primary infectious diseases. In addition, the potential neurological involvement should be considered to avoid persist impairments. The comprehensive medical history and physical examination should be conducted to find the subtle symptoms, as well as more specific scales [such as Composite Autonomic Symptom Score-31 for autonomic involvement (46)] and biochemical markers (such as neurofilament light chain protein). These suggestions are intended to enhance early detection and improve patient management.

The main limitations of our study are small sample sizes, selection bias, incomplete laboratory data and short-term follow-up. Only 53 participants were involved in this single-center retrospective analysis, and the medical records and laboratory data of some cases were incomplete. Therefore, although this study can provide valuable insights into neuroimmune manifestations of COVID-19, these observations must be validated through more rigorous study designs before generalizing the findings to a broader patient population. Long-term follow-up can assist in to understand the enduring effects of COVID-19-related neuroimmune complications.

## Conclusions

The manifestations of neuroimmune complications of COVID-19 are diverse and can manifest with severe neurological deficits early in the course of the disease. Clinicians should have prioritized immune assessments and initiate immunotherapy promptly upon exclusion of other etiologies to improve outcomes for patients with COVID-19 related neurological complications. Further research is required to validate these findings, elucidate the pathophysiological and molecular mechanism, as well as to evaluate the effectiveness of various immunomodulatory treatments.

## Data Availability

The original contributions presented in the study are included in the article/**Supplementary Material**. Further inquiries can be directed to the corresponding authors.
