# Supplementary material for: Clinical and immunological features in patients with neuroimmune complications of COVID-19 during Omicron wave in China: a case series
Source: Front Immunol. 2024 Dec 18;15:1499082. doi: 10.3389/fimmu.2024.1499082 (PMC11688395; doi:10.3389/fimmu.2024.1499082)
Supplement: Supplementary file 1 [file DataSheet1.docx]

| **Table. Clinical characteristics, managements and outcomes of all the patients with neuroimmune complications associated with COVID-19** | | | | | | | | | | | | | |
| --- | --- | --- | --- | --- | --- | --- | --- | --- | --- | --- | --- | --- | --- |
| Age/  Gender/  Classification | Confirmation of  SARS-CoV-2 | Onset  time | Neurological  symptoms | Imaging | Electrophy  -siology | CSF analysis | | | | Antibody of  SARS-CoV-2 | Cytokines  pg/ml | Auto-antibody | Managements＆Outcomes |
|  |  |  |  |  |  | WBC  (/ul) | TP  (mg/L) | | OCB |  |  |  |  |
| **GBS/CIDP** | | | | | | | | | | | | | |
| Case 1  66/M  **AMSAN** | Antigen test | 5d | Paraesthesia and weakness in 4 limbs | **Spinal cord** **MRI** (N) | **EMG**  Absent SNAP,  normal MCV,  prolonged F wave | 2 | | 1600 | I | N | Serum (N)  CSF (NM) | Serum  (anti-GD1b IgG, anti-GM1 IgG)  CSF  (anti-GD1b IgG) | IVIg  M mRS 4  90d mRS 2 |
| Case 2  55/F  **AIDP** | Antigen test | 14d | Progressive  symmetrical paralysis and paraesthesia of 4 limbs,  respiratory failure | **Spinal cord MRI** (NM) | **EMG**  Decreased MCV and SCV,  mild decreased amplitude of SNAP and CMAP,  prolonged latencies of F wave | 1 | | 180 | NM | NM | NM | Serum  (anti-NF186 IgG)  CSF  (anti-NF186 IgG) | IVIg  HDMP  PLEX  MV  M mRS 5  90d mRS 2 |
| Case 3  29/F  **CIDP** | 1^st^ RT-PCR  2^nd^ RT-PCR | 28d  12d | Recurrent tetraparesis | **Spinal cord** **MRI**  Contrast enhancement of L_5_, S_1_ spinal nerve roots | **EMG**  Prolonged distal  motor latencies,  decreased MCV | 1^st^ 1  2^nd^ 16 | | 1^st^ 946  2^nd^ 1680 | 1^st^NM  2^nd^ I | 1^st^NM  2^nd^ IgG | 1^st^NM  2^nd^  Serum (N)  CSF (NM) | 1^st^ NM  2^nd^  Serum (N) | 1^st^ IVIg  2^nd^ IVIg  HDMP  Tacrolimus  M mRS 4  90d mRS 0 |
| Case 4  44/F  **CIDP** | Antigen test | 21d | Paraesthesia in 4 limbs, weakness in lower limbs | **Spinal cord** **MRI** (N) | **EMG**  Decreased  MCV with conduction block,  prolonged latencies of F wave,  absent H reflex | 2 | | 1277 | I | IgG | Serum  (IL-6 16.5) | Serum(N) | IVIg  DXM  CTX  M mRS 4  90d mRS 1 |
| Case 5  60/M  **AIDP** | Antigen test | 6d | Weakness in 4 limbs and dysphagia | **Spinal cord** **MRI** (N) | **EMG**  Decreased  MCV,  absent F wave | 7 | | 1167 | NM | IgG | NM | Serum(N) | IVIg  M mRS 4  90d mRS 3 |
| Case 6  41/M  **AMSAN** | Antigen test | 21d | Paraesthesia in 4 limbs, dysphagia and dysarthria | **Spinal cord** **MRI** (N) | **EMG**  Decreased amplitude  of SNAP and CMAP,  absent H reflex | 2 | | 880 | I | NM | NM | Serum(N) | IVIg  M mRS 4  90d mRS 2 |
| Case 7  62/M  **CIDP** | RT-PCR | 3d | Diplopia, recurrent weakness and paraesthesia in 4 limbs | **Spinal cord** **MRI** (N) | **EMG**  Decreased  MCV with conduction block,  decreased amplitude of SNAP,  prolonged latencies of F wave,  absent H reflex | 9 | | 965 | I | NM | NM | Serum  (anti-GM4 IgG, anti-MAG IgM)  CSF  (anti-GD3 IgM, anti-GD2 IgM) | IVIg  HDMP  CTX  M mRS 3  90d mRS 1 |
| **Myelitis/spinal meningitis/NMOSD/MOGAD/ADEM** | | | | | | | | | | | | | |
| Case 8  36/F  **MOGAD** | Antigen test | 10d | Sensory changes up to T_8_, urinary  retention | **Spinal cord MRI**  Multifocal T2WI/FLAIR hyperintensity lesions (C_4-7_, T_5-6_)  **Brain MRI** (N) | **SEP**(N)  **VEP**  Prolonged latencies of bilateral P100 wave  **EEG**(N) | 18 | | 605 | I | IgG | Serum  (IL-2 6.10）  CSF  (TNF-α 25.3, IL-5 4.8, IL-6 131.3） | Serum  (anti-MOG IgG)  CSF  (anti-MOG IgG) | HDMP  IVIg  MMF  M mRS 3  90d mRS 1 |
| Case 9  55/F  **Spinal**  **meningitis** | Antigen test | 13d | Paraparesis, and sensory changes up to T_6_, urinary  retention | **Spinal cord**  **MRI**  Abnormal enhancement of the cervical spinal cord meninges | **SEP**  Absent  bilateral wave in the lower limbs | 2 | | 693 | I | IgG | Serum  (IL-2 6.10）  CSF (NM) | Serum(N)  CSF(N) | HDMP  PLEX  RTX  M mRS 4  90d mRS 4 |
| Case 10  38/F  **Myelitis** | Antigen test | 10d | Sensory changes up to T_10_ | **Spinal cord**  **MRI**  Small T2WI hyperintensity lesions with patchy enhancement (T_10_) | **SEP**  Prolonged latencies of P40 wave in the lower limbs | 7 | | 429 | I | NM | Serum(N）  CSF (NM) | Serum(N)  CSF(N) | HDMP  MMF  M mRS 3  90d mRS 1 |
| Case 11  13/F  **Myelitis** | Antigen test | 20d | Weakness in the right limb, and decreased sensation in the left limb | **Spinal cord**  **MRI**  Linear T2WI hyperintensity lesions (C_2_-_5_)  **Brain MRI**(N) | NM | 2 | | 160 | NM | NM | Serum(N）  CSF (NM) | Serum(N)  CSF(N) | HDMP  IVIg  MMF  M mRS 5  90d mRS 3 |
| Case 12  35/F  **Myelitis** | Antigen test | 2d | Paraparesis, and sensory changes up to T_10_, urinary  retention | **Spinal cord**  **MRI**  T2WI hyperintensity lesions(T_6_) | **SEP**  Decreased amplitude of right lower limb | 4 | | 166 | II | NM | NM | Serum(N)  CSF(N) | HDMP  M mRS 3  90d mRS 0 |
| Case 13  58/M  **MRI negative**  **myelitis** | Antigen test | 2d | Paraparesis, and sensory changes up to T_3_, urinary  retention | **Spinal cord**  **MRI**(N)  **Brain MRI** (N) | NM | 20 | | 824 | NM | NM | Serum(N)  CSF  (IL-8 495, IL-6 6.7, IL-5 5.2) | Serum (N)  CSF(N) | DXM  IVIg  IA  M mRS 5  90d mRS 4 |
| Case 14  60/F  **Myelitis** | Antigen test | 10d | Paraparesis, and decreased sensation in lower limbs | **Spinal cord**  **MRI**  T2WI hyperintensity lesions(T_10-11_) | **SEP**  Prolonged latencies of P40 wave in the lower limbs | 2 | | 400 | I | IgG  IgM | Serum(N)  CSF(NM) | Serum(N)  CSF(N) | HDMP  MMF  M mRS 3  90d mRS 3 |
| Case 15  59/F  **Spinal**  **meningitis** | Antigen test | 4d | Paraparesis, decreased sensation in lower limbs, and bowel and bladder dysfunction | **Spinal cord**  **MRI**  Linear enhancement at the conus medullaris | **SEP**  (N) | 12 | | 430 | I | IgG | Serum  (IL-1β 17.3)  CSF(NM) | Serum(N)  CSF(N) | HDMP  M mRS 4  90d mRS 2 |
| Case 16  38/F  **NMOSD** | Antigen test | 21d | Numbness in both upper limbs | **Spinal cord**  **MRI**  Longitudinally extensive T2WI hyperintensity lesions(C_1-6_) | **SEP**  Prolonged latencies of bilateral N13 wave and left N20 in the upper limbs  **VEP**(N)  **EEG**(N) | 5 | | 450 | I | IgG | Serum(N)  CSF(NM) | Serum  (anti-AQP4 IgG)  CSF(NM) | HDMP  MMF  Inerizumab  M mRS 2  90d mRS 1 |
| Case 17  77/M  **Myelitis** | RT-PCR | 3d | Paraparesis, decreased sensation in lower limbs, and bowel and bladder dysfunction | **Spinal cord**  **MRI**  Longitudinally extensive T2WI hyperintensity lesions(T_8_-L_2_) | **SEP**  Prolonged latencies of bilateral N13 and N20 wave, absent bilateral wave in the lower limbs | 8 | | 410 | IV | NM | Serum (N)  CSF（NM） | Serum(N)  CSF(N) | HDMP  DXM  CTX  M mRS 4  90d mRS 4 |
| Case 18  66/M  **MRI negative**  **myelitis** | RT-PCR  Antigen test | 12d | Paraparesis, decreased sensation in lower limbs and bladder dysfunction | **Spinal cord**  **MRI**(N) | **SEP**  Prolonged latencies of bilateral wave in the upper and  lower limbs | 4 | | 356 | I | IgG | Serum(N)  CSF  (IL-8 128, IL-5 4.0, IL-6 5.8, TNF-α 20.6) | Serum(N)  CSF(N) | DXM  M mRS 4  90d mRS 3 |
| Case 19  36/M  **Myelitis** | RT-PCR | 5d | Paraparesis in  lower limbs and bladder dysfunction | **Spinal cord**  **MRI**  T2WI hyperintensity lesions(T_6-9_) | **SEP**  Prolonged latencies of bilateral wave in the upper and  lower limbs | 1 | | 900 | II | NM | Serum(NM)  CSF（NM） | Serum(N)  CSF(N) | HDMP  PLEX  MMF  M mRS 4  90d mRS 2 |
| Case 20  64/F  **MRI negative**  **myelitis** | RT-PCR | 5d | Paraparesis, decreased sensation in lower limbs and bladder dysfunction | **Spinal cord**  **MRI** (N) | **SEP**  Prolonged latencies of P40 wave in the lower limbs | 9 | | 515 | NM | NM | Serum  (IL-6 N)  CSF (N) | NM | DXM  IVIg  M mRS 4  90d mRS 2 |
| Case 21  56/F  **NMOSD** | RT-PCR | 30d | Hiccups, paraparesis in upper and lower limbs, and decreased sensation in lower limbs | **Spinal cord**  **MRI**  T2WI hyperintensity lesions with patchy enhancement (C_2-3_,C_6_-T_1_)  **Brain MRI**  T2WI /FLAIR hyperintensity lesions in posterior horn of lateral ventricle and medulla oblong | **SEP**  Prolonged latencies of bilateral wave in the upper and  lower limbs  **VEP**(N)  **EEG**(N) | 41 | | 1140 | I | NM | Serum(N)  CSF(NM) | Serum  (anti-AQP4 IgG)  CSF(NM) | HDMP  CTX  M mRS 5  90d mRS 3 |
| Case 22  62/M  **ADEM** | Antigen test | 7d | Tremor, unsteady gait, and bowel and bladder dysfunction | **Spinal cord**  **MRI**  Longitudinally extensive T2WI hyperintensity lesions(cervical and thoracic spinal cord)  **Brain MRI**  T2WI /FLAIR hyperintensity lesions in bilateral- limbic lobe, periventricular, around the third ventricle and adjacent to the aqueduct of the midbrain regions | **SEP**  Prolonged latencies of bilateral wave in the upper and  lower limbs  **VEP**(N)  **EEG**(N) | 21 | | 2230 | II | NM | NM | Serum(N)  CSF(N) | HDMP  IVIg  M mRS 3  90d mRS 1 |
| Case 23  20/M  **MOGAD** | Antigen test | 6d | Vision lost in the left eye, neck pain, paraparesis, decreased sensation in lower limbs and bladder dysfunction | **Brain MRI**  Patchy T2WI /FLAIR hyperintensity, DWI iso-intensity lesions(left frontal lobe, cerebral peduncles, bilateral limbic lobes)  **Spinal cord**  **MRI**  Patchy T2WI hyperintensity lesions in spinal cord | **VEP**  Prolonged latencies of left P100 wave  **EEG**  Increased theta activity  **SEP**(N) | 132 | | 996 | I | IgG | Serum(N)  CSF(NM) | Serum  (anti-MOG IgG)  CSF(NM) | HDMP  IVIg  M mRS 3  90d mRS 1 |
| **AIE** | | | | | | | | | | | | | |
| Case 24  15/F  **Anti-Ri** **encephalitis ** | Antigen test | 10d | Psychiatric symptom | **Brain MRI**  DWI cortex hyperintensity in bilateral frontal and temporal lobe  **PET/CT** Reduced FDG uptake in left temporal lobe,  TSPO (N) | **EEG**  Background  slowing | 1  NGS(NM) | | 310 | I | IgG | Serum (N)  CSF (N) | Serum  (anti-Ri IgG)  CSF(N) | IVIg  HDMP  MMF  M mRS 3  90d mRS 0 |
| Case 25  17/M  **Anti-GlyR1 encephalitis** | RT-PCR | 7d | Psychiatric symptom | **Brain MRI**  (N)  **PET/CT**  FDG-PET  Increased FDG uptake in bilateral putamen,  reduced FDG uptake in the remaining cortex.  TSPO-PET (N) | **EEG**(NM) | 0  NGS  HSV-1 3c | | 634 | I | IgG | Serum (N)  CSF  (IL-8 100.8) | Serum  (anti-GlyR1 IgG)  CSF  (anti-GlyR1 IgG) | IVIG  HDMP  RTX  M mRS 3  90d mRS 0 |
| Case 26  17/F  **Anti-NMDAR encephalitis** | Antigen test | 3d | Psychiatric symptom and seizure | **Brain MRI**  (N)  **PET/CT**  FDG-PET  Reduced FDG uptake in bilateral parieto-occipital and temporal lobes, right thalamus and left cerebellum.  TSPO-PET  Mild elevation of TSPO binding in the bilateral medial temporal lobes | EEG  Increased delta and theta activity | 9 | | 270 | I | IgG | NM | Serum  (anti-NMDAR IgG)  CSF  (anti-NMDAR IgG) | HDMP  IVIG  MMF  PLEX  M mRS 3  90d mRS 0 |
| Case 27  6/F  **MNOS** | RT-PCR | 28d | Vision loss, walk and consciousnes-s disorders | **Brain MRI**  FLAIR hyperintensity lesions in right optic nerve, cerebellum, pons and thalamus  Spinal cord MRI(N) | EEG  Increased delta activity in  occipital region | 77  NGS  EBV 11c | | 436 | II | NM | Serum (IFN-γ23.7)  CSF(NM) | Serum  (anti-MOG IgG)  CSF  (anti-NMDAR IgG) | HDMP  IVIG  MMF  Antiepileptic drug  M mRS 3  90d mRS 0 |
| Case 28  10/F  **Anti-NMDAR encephalitis** | RT-PCR | 7d | Fever, headache, seizure and central hypoventilation | **Brain MRI** (N)  **PET/CT**  FDG-PET  Decreased FDG uptake in bilateral cerebral  cortex, especially in right parieto-occipital temporal lobe and cingulate gyrus  Increased FDG uptake in bilateral basal ganglia  TSPO-PET (N) | EEG  Sharp waves,  spike-and-slow waves and diffuse slowing activity | NM  NGS  EBV  11c | | NM | NM | NM | NM | CSF  (anti-NMDAR IgG) | HDMP  IVIG  RTX  MMF  Antiepileptic drug  Mechanical ventilation  M mRS 5  90d mRS 1 |
| Case 29  88/F  **anti-GABAbR encephalitis** | RT-PCR | 4d | Fever and seizure | **Brain MRI** (N)  **PET/CT**  Decreased FDG uptake in bilateral cerebral  cortex ( bilateral temporo-parietal lobes and left frontal lobe ) | EEG  Increased delta and theta ac-  tivity | 1  NGS(N) | | 410 | IV | IgG | Serum  (IL-6 15.4, IFN-γ 31.4)  CSF  (IL-8 142.4) | Serum  (anti-GABA_b_R IgG)  CSF(N) | MP  IVIG  MMF  Paxlovid  Antiepileptic drug  Anticoagula-nt drug  M mRS 3  90d mRS 1 |
| Case 30  53/F  **Limbic**  **encephalitis** | RT-PCR | 14d | Fever, psychiatric symptom | **Brain MRI**  Patchy T2WI /FLAIR hyperintensity lesions(bilateral temporal lobes) | NM | 1  NGS(N) | | 412 | NM | NM | Serum(N)  CSF(NM) | Serum(N)  CSF(N) | HDMP  IVIg  M mRS 2  90d mRS 0 |
| Case 31  52/M  **Antibody-negative AIE** | RT-PCR | 9d | Fever, consciousness and psychiatric disorders | **Brain MRI**  (N)  **PET/CT**  Reduced FDG uptake in bilateral frontal, parietal, occipital, and temporal lobes, mild elevation of TSPO binding in the medial aspect of bilateral temporal lobes | **EEG**  Increased theta activity and sharp waves | 15  NGS(NM) | | 1081 | II | IgG | Serum(NM)  CSF(NM) | Serum(N)  CSF(N) | HDMP  IVIg  LMWH  Antiepilepti-c therapy  M mRS 5  90d mRS 0 |
| Case 32  65/F  **Limbic**  **encephalitis** | RT-PCR | 3d | Fever, consciousness and psychiatric disorders | **Brain MRI**  Gyrus-like T2WI /Flair hyperintensity lesions with swelling cortex(left temporal). | **EEG**  Slowing  background, increased theta activity and sharp wave | 12  NGS(NM) | | 2512 | I | IgG | NM | Serum(N)  CSF(N) | DMX  Ganciclovir  Azvudine  Antiepileptic  therapy  M mRS 5  90d mRS 1 |
| Case 33  53/M  **Limbic**  **encephalitis** | RT-PCR | 2d | Fever, psychiatric and cognitive disorders | **Brain MRI** T2WI /FLAIR hyperintensity lesions in bilateral hippocampus.  **PET/CT**  Reduced FDG uptake in bilateral cerebral and cerebellar cortex  TSPO PET (N) | **EEG**  Increased theta activity | 9  NGS(N) | | 660 | V | NM | NM | Serum(N)  CSF(N) | IVIG  MP  M mRS 3  90d mRS 1 |
| **Other encephalopathy/encephalitis** | | | | | | | | | | | | | |
| Case 34  67/F | Antigen test | 7d | Psychiatric symptom | **Brain MRI** (N) | **EEG**  Increased theta and delta activities | 0  NGS(NM) | | 420 | I | NM | Serum  (IL-6 11.4)  CSF(NM) | Serum(N)  CSF(N) | MP  Ganciclovir  M mRS 5  90d mRS 1 |
| Case 35  15/M | Antigen test | 4d | Psychiatric symptom | **Brain MRI**(N) | **EEG**  Increased theta activity | 12  NGS(N) | | 460 | I | NM | NM | Serum(N)  CSF(N) | HDMP  Antiepileptic therapy  M mRS 3  90d mRS 0 |
| Case 36  19/F  **Brainstem encephalitis** | Antigen test | 3d | Ataxia, eye movement disorder | **Brain MRI** (N) | **EEG**  Increased theta and delta activities, and sharp wave | 1  NGS(NM) | | 455 | I | NM | NM | Serum(N)  CSF(N) | MP  IVIg  M mRS 5  90d mRS 2 |
| Case 37  26/F | RT-PCR  Antigen test | 2d | Psychiatric disorder and seizure | **Brain MRI**  T2WI /FLAIR hyperintensity lesions in bilateral thalamus, temporal lobe, hippocampus and corpus callosum  **PET/CT**  Reduced FDG uptake in bilateral frontal, temporal and occipital lobes and cerebellum,  Increased FDG uptake in parietal lobe, posterior cingutate and motor cortex | **EEG**  Increased theta activity | 0  NGS(NM) | | 660 | I | NM | NM | Serum(N)  CSF(N) | HDMP  IVIg  PLEX  Antiepileptic therapy  M mRS 4  90d mRS 3 |
| Case 38  64/F | Antigen test | 3d | Psychiatric disorder, cognitive disorder and seizure | **Brain MRI**  T2WI /FLAIR hyperintensity lesions in bilateral frontotemporal parietal cortex | **EEG**  Increased theta activity and sharp wave | 2  NGS(N) | | 540 | I | NM | Serum  (IL-6 12.42) | Serum(N)  CSF(N) | HDMP  IVIg  Antiepileptic therapy  Mechanical ventilation  M mRS 5  90d mRS 5 |
| Case 39  51/F | Antigen test | 2d | Cognitive disorder and ataxia | **Brain MRI**  T2WI /FLAIR hyperintensity lesions in pons, bilateral hippocampus, thalamus, basal ganglia  **PET/CT** Reduced FDG uptake in bilateral frontal lobes and cerebellum, increased FDG uptake in bilateral putamen, mild elevation of TSPO binding in the right temporal lobe | **EEG**  Increased theta and delta activities | 8 | | 1110 | I | NM | Serum  (IL-1β 34.3, IL-5 3.6)  CSF(NM) | Serum(N)  CSF(N) | HDMP  IVIg  M mRS 4  90d mRS 3 |
| Case 40  75/F | RT-PCR | 3d | Fever, psychiatric and consciousness disorders | **Brain MRI** T2WI /FLAIR hyperintensity lesions in thalamus, bilateral basal ganglia, paraventricular and frontoparietal lobes | **EEG**  Increased theta and delta activities | 123  NGS(NM) | | 1100 | I | NM | NM | Serum(N)  CSF(N) | HDMP  PLEX  Anticoagula-nt and  antibacterial therapy  M mRS 5  90d mRS 4 |
| Case 41  16/M | Antigen test | 0d | Psychiatric and consciousness disorders | **Brain MRI** T2WI /FLAIR hyperintensity lesions in pons, bilateral temporal lobes and cingulate cortex.  **PET/CT**  Reduced FDG uptake in bilateral parietal, temporal and cerebellar cortex  **TSPO PET** (N) | **EEG**(NM) | 20  NGS(N) | | 567 | I | NM | NM | Serum(N)  CSF(N) | HDMP  IVIG  M mRS 4  90d mRS 0 |
| Case 42  7/F | RT-PCR | 5d | Fever, consciousness disorder  and seizure | **Brain MRI**  Symmetry T2WI /FLAIR hyperintensity lesions in bilateral external bursae, hippocampus, temporal horn of lateral ventricle, and paraventricular region | **EEG**  Slowing  background | 7  NGS(2019-nCOV 2c) | | 421 | I | NM | NM | Serum(N)  CSF(N) | HDMP  IVIG  Antiepileptic therapy  Mechanical ventilation  M mRS 5  90d mRS 2 |
| Case 43  68/M | Antigen test | 14d | Cognitive disorder | **Brain MRI**  T2WI /FLAIR hyperintensity lesions in bilateral paraventricular region | **EEG**  Slowing  background, diffuse theta and delta activities | 13 | | 540 | I | IgG | Serum  (IL-1β 44.2, IL-5 4.3, IL-8 24.0)  CSF(NM) | Serum(N)  CSF(N) | MP  IVIG  M mRS 5  90d mRS 2 |
| Case 44  31/M | RT-PCR | 17d | Cognitive and psychiatric disorders | **Brain MRI**  T2WI /FLAIR hyperintensity lesions in bilateral globus pallidus, paraventricular region, centrum semiovale and corona radiata | **EEG**  Increased theta and delta activities | 21 | | 474 | NM | NM | NM | Serum(N)  CSF(N) | MP  Hyperbaric oxygen  M mRS 3  90d mRS 1 |
| Case 45  35/F | RT-PCR | 14d | Fever, headache and psychiatric disorder | **Brain MRI**  Symmetrical FLAIR hyperintensity lesions in bilateral basal ganglia  **PET-CT**  Decreased FDG uptake in bilateral frontal lobes and cerebellum.  Increased FDG uptake in bilateral putamen  TSPO-PET (N) | **EEG**  Background  slowing | 10  NGS(N) | | 1180 | I | IgG | Serum  (IL-1 6.35, IL-8 104.0)  CSF(N) | Serum(N)  CSF(N) | MP  IVIg Ganciclovir  RTX  MMF  M mRS 4  90d mRS 1 |
| Case 46  18/M | Antigen test | 3d | Psychiatric disorder | **Brain MRI**  (N)  **PET/CT**  FDG-PET  (N)  TSPO-PET  elevation of TSPO binding in the bilateral medial temporal lobes | **EEG**  (N) | 0  NGS(N) | | 410 | I | IgG | Serum (NM)  CSF  (IL-8 79.4) | Serum(N)  CSF(N) | HDMP  IVIG  M mRS 3  90d mRS 0 |
| Case 47  61/F | RT-PCR | 7d | Reduced walking ability and cognitive disorder | **Brain MRI**  (N) | **EEG**(NM) | 210 | | 530 | I | NM | NM | Serum(N)  CSF(N) | MP  PLEX  IVIG  M mRS 5  90d mRS 6 |
| Case 48  15/F  **ANE** | RT-PCR | 2d | Fever and consciousness disorder | **Brain MRI**  FLAIR hyperintensity lesions in thalamus, hippocampus, brainstem and  SWI detecting cerebral hemorrhage in above lesions | **EEG**  Background  slowing with increased theta activity | 2  NGS(N) | | 2141 | I | NM | Serum  (IL-6 23.18)  CSF(NM) | Serum(N)  S-TBA(P)  CSF(N)  C-TBA(N) | DXM  IVIG  Antiepileptic drug  Anticoagula-nt drug  Paxlovid  hyperbaric oxygen  M mRS 5  90d mRS 3  L mRS 1 |
| Case 49  44/M | RT-PCR | 0d | Mental and consciousness disorders | **Brain MRI**  (N) | **EEG**  Slowing background with paroxysmal30  sharp and sharp slow waves | 1 | | 569 | I | IgG | NM | Serum(N)  CSF(N) | DXM  Ganciclovir  M mRS 4  90d mRS 0 |
| Case 50  70/F | RT-PCR | 1d | Fever, consciousness disorder and headache | **Brain MRI**  (N) | **EEG**  Slowing background with paroxysmal sharp and sharp slow waves | 2  NGS(N) | | 551 | I | N | Serum (N) | NM0 | DXM  IVIG  M mRS 3  90d mRS 0 |
| **Cerebellitis** | | | | | | | | | | | | | |
| Case 51  30/M | Antigen test | 9d | Gait and speech ataxia | **Brain MRI**  **(N)** | **EEG**(NM) | 15 | | 320 | I | NM | NM | Serum(N)  CSF(N) | DXM  IVIG  M mRS 4  90d mRS 0 |
| Case 52  18/M | RT-PCR | 2d | Gait and speech ataxia | **Brain MRI**  FLAIR hyperintensity lesions in bilateral cerebellum | **EEG**(NM) | 10  NGS(N) | | 789 | I | NM | NM | Serum(N)  CSF(N) | HDMP  MMF  M mRS 4  90d mRS 2 |
| Case 53  59/F | RT-PCR | 3d | Gait and speech ataxia | **Brain MRI**  FLAIR hyperintensity lesions in bilateral cerebellum | **EEG**(NM) | 9 | | 574 | I | NM | Serum  (IL-1β 25.5, IL-5 5.1)  CSF(N) | Serum(N)  CSF(N) | MP  hyperbaric oxygen  M mRS 4  90d mRS 3 |
| CSF= cerebrospinal fluid, WBC= white blood cell, TP=total protein, OCB= oligoclonal bands, MRI= magnetic resonance imaging, AMSAN= acute motor-sensory axonal neuropathy, EMG= Electromyography, SNAP= sensory nerve action potential, MCV= motor nerve conduction velocity, N= negative, NM= not mentioned, IVIg= intravenous immunoglobulin, M mRS= maximal modified Rankin scale, 90d mRS= 90 day modified Rankin scale, AIDP= acute inflammatory demyelinating polyradiculoneuropathy, SCV= sensory nerve conduction velocity, CMAP= compound muscle action potential, HDMP= high-dose methyl prednisolone, PLEX= plasma exchange, CIDP= chronic inflammatory demyelinating polyneuropathy, RT-PCR= reverse transcription-polymerase chain reaction, IL= Interleukin, DXM= dexamethasone, CTX= cyclophosphamide, MOGAD= myelin oligodendrocyte glycoprotein antibody-associated disease, MOG= myelin oligodendrocyte glycoprotein, FLAIR= fluid-attenuated inversion-recovery sequency, SEP= somatosensory evoked potential, VEP= visual evoked potential, EEG= electroencephalography, TNF= tumor necrosis factor, MMF= mycophenolate mofetil, RTX= rituximab, IA= immunoadsorption, NMOSD= neuromyelitis optica spectrum disorders, AQP4= aquaorin-4, ADEM= acute disseminated encephalomyelitis, AIE= autoimmune encephalitis, DWI= diffusion weighted imaging, NGS= next generation sequencing, PET/CT= positron emission tomography/computed tomography, FDG= fluorodeoxyglucose, TSPO= translocator protein, GlyR= Glycine receptor, HSV-1= Herpes simplex virus type 1, NMDAR= N-methyl-D-aspartic acid receptor, MNOS= the overlapping syndrome of MOG-antibody disease and NMDAR encephalitis, EBV= epstein-barr virus, IFN= interferon, GABA_b_R= gamma-aminobutyric acid type B receptor, LMWH= low molecular weight heparin, MP= methyl prednisolone, ANE= acute necrotizing encephalopathy | | | | | | | | | | | | | |
